# Potential application of hydrogel to the diagnosis and treatment of multiple sclerosis

**DOI:** 10.1186/s13036-022-00288-7

**Published:** 2022-04-08

**Authors:** Haochuan Liu, Bing Chen, Qingsan Zhu

**Affiliations:** 1 Department of Orthopaedics, China-Japan Union Hospital of Jilin University, Xiantai Street No. 126, Changchun, TX 130031 PR China; 2 Department of Anesthesiology, China-Japan Union Hospital of Jilin University, Xiantai Street No. 126, Changchun, TX 130031 PR China

**Keywords:** Hydrogel, Multiple sclerosis, Biosensor, Cell culture, Cell delivery

## Abstract

**Abstract:**

Multiple sclerosis (MS) is a chronic demyelinating disease of the central nervous system. This disorder may cause progressive and permanent impairment, placing significant physical and psychological strain on sufferers. Each progress in MS therapy marks a significant advancement in neurological research. Hydrogels can serve as a scaffold with high water content, high expansibility, and biocompatibility to improve MS cell proliferation in vitro and therapeutic drug delivery to cells in vivo. Hydrogels may also be utilized as biosensors to detect MS-related proteins. Recent research has employed hydrogels as an adjuvant imaging agent in immunohistochemistry assays. Following an overview of the development and use of hydrogels in MS diagnostic and therapy, this review discussed hydrogel’s advantages and future opportunities in the diagnosis and treatment of MS.

**Graphical abstract:**

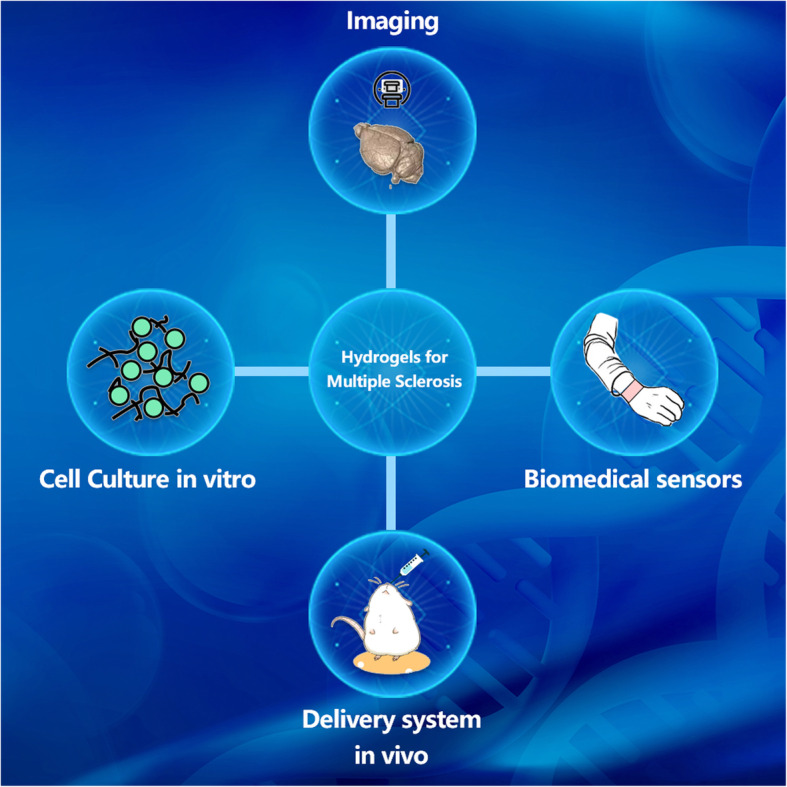

## Background

Trauma, stroke, neurodegenerative or demyelinating diseases, spinal cord injury, traumatic brain injury, Alzheimer’s disease, Parkinson’s disease, and multiple sclerosis (MS) may damage the central nervous system (CNS). MS is a chronic inflammatory disease characterized by perivenous lesions that eventually result in demyelinating plaques, oligodendrocyte destruction, and irreparable damage to gray and white matter axons [1, 2]. The range of MS comprises radiologically isolated syndrome (RIS), clinically isolated syndrome (CIS), and clinically diagnosed MS. Clinically diagnosed MS may fall into the following categories based on the various aspects of the disease course, which are relapsing-remitting multiple sclerosis (RRMS as the most common type of MS), secondary progressive multiple sclerosis (SPMS), primary progressive multiple sclerosis (PPMS), and progressive relapsing multiple sclerosis (PRMS, the rarest in the course of MS) [3]. MS is diagnosed using a combination of clinical, radiographic, and laboratory data, including the patient’s history, cerebrospinal fluid (CSF) examination, oligoclonal bands (OCB), immunoglobulin G (igG synthesis), visual performance potential (VEP), and magnetic resonance imaging (MRI) [4, 5]. Additionally, MS diagnosis necessitates a distinction between idiopathic inflammatory disorders, such as neuromyelitis optica spectrum disorder, and other recurrent diseases that mirror MS. Despite this, MS may still be misdiagnosed owing to certain deceptive imaging findings, resulting in around a quarter of individuals receiving MS therapy being diagnosed with another disease [6]. Anti-inflammatory and anti-immune treatments are the primary therapies for MS. The most often utilized therapies are adrenal glucocorticoids, immunosuppressor such as cyclophosphamide, azathioprine, and cyclocytosine A, plasmapheresis, immunoglobulin, and immunomodulators such as β-interferon, copolymer-1, and others [7, 8]. However, the effectiveness is less than optimal. Additionally, new therapeutics such as stem cells need ongoing management of cell selection, transplantation modality, and differentiation direction [9]. Despite significant advancements in the diagnosis and treatment of MS, current approaches remain restricted, and new ones are needed. One of the more intriguing solutions under investigation is the use of hydrogel-based biomaterials. Hydrogels have played an increasingly essential role in the biomedical area in various ways, including combinations due to their growing complexity of function and structure [10–14]. In the field of neurodegenerative diseases such as multiple sclerosis, the entire process of diagnosis and treatment research, from examination to drug administration, cell transplantation, in vitro disease simulation, and even imaging to monitor disease progression, requires the collaboration of medical and industrial disciplines. Hydrogel, being an exceptional representation of bioengineering materials with superior performance, may perform well in all of the aforementioned linkages. A hydrogel is a three-dimensional network polymer with hydrophilic structures that absorb thousands of times its dry weight in water. Additionally, hydrogels exhibit highly adjustable physical characteristics, including degradability, mechanical strength, gelation duration, and gelation temperature [11, 15]. Hydrogels have been extensively employed in biosensing, cell encapsulation, drug delivery, and tissue engineering scaffolds, among other applications, including but not limited to the area of neurodegenerative disease [16, 17].

Hydrogels may be classified according to their origin (natural or synthetic), composition (homopolymer or copolymer), reaction circumstances, the crosslinking mechanism (chemical or physical crosslinking), and charge. Many publications and reviews are available on the synthesis, characteristics, and uses of hydrogels, and the following review is highly suggested for interested readers [18–25]. The recent decade has witnessed advancements in mixing diverse components and methods to prepare hydrogel with enhanced properties. These novel excellent physicochemical qualities have established hydrogels as advanced biomaterials, prompting a host of research on their ultimate translation into therapeutic applications [26]. Hydrogels made of diverse materials are extensively utilized; only a few have been studied in MS. These hydrogels have the potential to play a critical role in the diagnosis and treatment of MS, such as supporting the growth of MS-related cells, improving the delivery of therapeutic agents including cells in vivo. Hydrogels may also be utilized as biosensors to detect MS-related biomolecule, and recent research have shown that hydrogels can be employed as auxiliaries in immunohistochemistry investigations to aid in imaging, and other aspects. This review primarily covered the use of hydrogel in the diagnosis and treatment of MS aims to generate new ideas and references for the confluence between MS diagnostic and treatment and hydrogel biomaterials.

## Multiple sclerosis

Chronic inflammation of the brain and spinal cord, demyelination, and neurodegeneration are the symptoms of MS, which diminishes the patient’s physical performance progressively [27]. MS affects an estimated 2.5 million individuals globally, and its incidence has grown in recent decades [28, 29]. Gender could impact the morbidity of MS: women are more vulnerable than men, with a female-to-male morbidity ratio ranging between 2:1 and 3:1 depending on the geographic area [30]. Also, the unequal frequency distribution suggests that both environmental and genetic variables contribute significantly to MS progression [31]. Several identified environmental risk factors include Epstein-Barr virus (EBV) infection, teenage obesity, smoking, insufficient vitamin D, and sun exposure [32]. Most research on genetic factors focuses on the human leukocyte antigen (HLA) region on the short arm of chromosome 6 (6P21). Changes in this area may be positively or negatively associated with disease risk and course [33]. MS is caused by a combination of environmental, genetic, and epigenetic factors, which may combine with various identified modifiable risk factors [34]. MS pathological markers include demyelination across the blood-brain barrier by autoreactive T and B cells, neuronal and axonal damage and loss, and astrocyte growth [35, 36]. In severe MS, axonal damage progresses slowly to axonal transection inside demyelinated plaques, eventually culminating in irreparable damage [36, 37]. However, the mechanism by which the immune response to CNS antigens is initiated and maintained in MS remains unexplained. MS in its early stages is often characterized by an immediate onset of recurring neurological impairments, depending on the area of the central nervous system affected by the acute inflammatory demyelinating disease and the inflammatory process severity. Some significant symptoms include but are not limited to optic neuritis, tremor, nystagmus, slurred speech, incoordination and gait instability, limb numbness or weakness, weariness, subacute motor loss, diplopia, and discomfort [38, 39]. McDonald’s criteria for MS amended in 2017 reinstated the significance of cerebrospinal fluid abnormalities in the diagnosis [40]. On the other hand, it has been proposed that standardized MRI methods be used to evaluate individuals with suspected or clinically proven MS [41]. MS therapy may be classified into two groups: symptomatic treatments and disease-modifying therapies (DMT) that seek to change the disease’s course, and the route of administration classifies DMT into self-injection, oral or intravenous preparations [42]. Improvements in MS therapy are unparalleled in any other field of neurology, and stem cell treatment and immunotherapy offered promise, but there has been no “magic bullet” capable of entirely curing the condition, which is currently deemed incurable [43–48]. The priority now is to limit the disease’s effect throughout the disease course, enhance the quality of life, and promote a healthy philosophy for MS patients [34, 49].

## Hydrogels applied in MS and its characteristics

### Biosensing

Hydrogels are increasingly being utilized to fabricate electrochemical sensors, and researchers are starting to investigate their biological applications [50]. Electrochemical sensors are a kind of sensor in which a sensing element interacts with the target analyte to produce a sensing signal. For qualitative or quantitative investigation, these specialized sensors transform data to recognizable electrical signals proportional to the concentration of the target analyte [51]. Due to the unique microwater environment created by hydrogels, they may function as substrates for biomolecules to sustain their biological activity. Simultaneously, hydrogels have a very high specific surface area owing to their three-dimensional structure. As a result, hydrogels are primarily exploited in electrochemical sensor research as a substrate for immobilizing biomolecules [52]. These biomolecular hydrogels are capable of identifying analytes with high specificity, which is very useful in the diagnosis of MS.

Matrix metalloproteinase-9 (MMP-9) is a significant peripheral biomarker of neuroinflammation in MS [53, 54]. In the diagnosis and detection of MS, the measurement of protease level by the electrochemical sensor may eliminate the tedious methods of biochemical analysis such as ELISA and reduce the personnel and financial cost of detecting instruments such as MRI. More crucially, it can not be confined by the monitoring environment [55, 56]. Many electrochemical sensors with various identification components have been developed, including immune or active sensors. A disposable biosensor monitoring the degradation of hydrogel films is a promising platform capable of monitoring protease activity, which is small, affordable, and simple to operate and has the potential of mass manufacturing [57]. Biela et al. [58] synthesized it by coating electrodes with oxidized-dextran and then cross-linking with peptides having particular cleavage sites of MMP-9. Exposure to enzymes induces film degradation, which may be tracked via impedance measurements. Results indicated effective detection of MMP-9 in the clinically relevant range of 50 to 400 ng/ ml. Except for reacting within 5 min, the sensor exhibited high selectivity to MMP-9 in the presence of MMP-2. However, reaction delays at low enzyme concentration and low stability of quartz crystal microbalance (QCM) signal before introducing enzyme existed. Ahmad et al. further employed poly (2-oxazoline) crosslinked with protease-specific lytic peptides as raw materials to construct hydrogel membranes on gold-plated quartz crystals using thiol-ene click chemistry and improve the crosslinking density. They measured the degradation rate of the hydrogel using a quartz crystal microbalance (QCM), which indicated a considerable dependence on MMP-9 concentration. Experiments examined the concentration range of 0–160 nM MMP-9 and identified the detection limit of 10 nM MMP-9 [59]. In addition, the materials utilized to build disposable MMP-9 sensors are universal and can detect various proteases by modifying the peptide sequence.

**Fig. 1:**
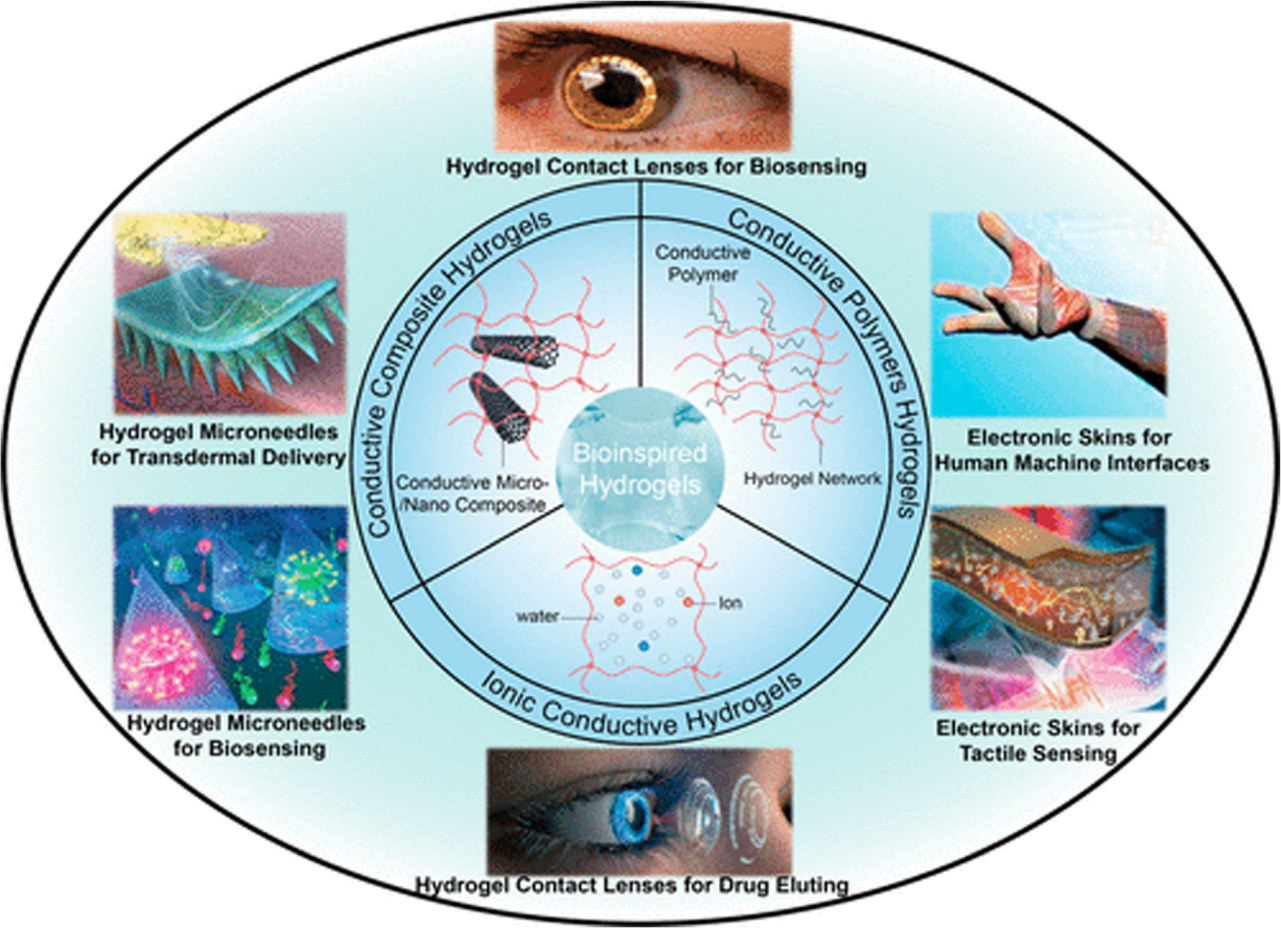
Bionic hydrogel materials and their applications in recently developed wearable devices [60]. Copyright 2021 American Chemical Society

On the other hand, hydrogels have been extensively studied for their multiple functions in wearable devices due to their exceptional flexibility, inherent electrical conductivity, biocompatibility, and rapid stimulus-response, as well as their unique mechanical properties (excellent stretchability, adjustable toughness, and low elastic modulus), see Fig. 1 [61]. Wearable devices can quantify biochemical analytes, monitor physiological parameters, detect human movement, and interact with external environmental stimuli. Sensing devices that monitor physiological signals and quantify disease biomarkers are critical for the early detection and intervention of neurodegenerative illnesses, as well as for the administration of medicine and correct evaluation of treatment effects [62]. The present need for bio sensing systems that can detect physiological signals consistently and precisely, as well as biocompatible surface chemistry and device-human interface interactions, is driving continuous research into enhanced sensing materials, sensing techniques, and device designs [63]. Hydrogels are hydrophilic polymers that contain a significant amount of water and so resemble human tissue. They may not only serve as polymer substrates for the loading of functional materials for biological signal transduction, but can also react to stimuli in conjunction with filling materials to further improve sensing performance [64].

Bionic hydrogels employed in wearable devices and biosensing include ionic conductive hydrogels, conductive polymer hydrogels, and conductive micro/nanocomposite hydrogels [60]. Materials and manufacturing processes may enable hydrogel with varying ionic and electrical conductivity, biocompatibility, biodegradability, antibacterial activity, self-healing and injectability, self-viscosity, transparency, and long-term stability extensibility, compressibility, and fatigue resistance, among other properties [65–68]. Currently, wearable technologies in MS are primarily used to monitor mobility and balance, and they may eventually play a more prominent role in assessing tiredness, tremor, and spasm [69, 70]. Wearable devices may give critical information for tracking the course of MS and evaluating the therapeutic benefits of disease-modifying and symptomatic medications [69, 71]. Despite few examples of hydrogel-type wearable sensors being explicitly used for MS, they have been utilized to detect changes in neurophysiological activity such as tremors in neurodegenerative disorders such as Parkinson’s disease [72]. Many aspects impacting the quality of life of multiple MS are expected to be simply and accurately recorded with the development of hydrogel-related wearable devices and biosensing.

In summary, these hydrogels and their composites are increasingly enabling the detection of neurodegenerative disease biomarkers, physiological signals, and macroscopic symptom presentations such as MS. Simultaneously, hydrogel sensors provide some benefits in terms of repeatability and quality guarantee period [73]. Hydrogels combined with various functional materials and device designs are expected to provide wearable or implantable multifunctional healthcare platforms capable of diagnosis and treatment in the future.

### In vitro cell culture

MS is a degenerative demyelinating disease of the CNS. One of the most significant unresolved issues in MS research is the development of neuroprotective and myelin regeneration strategies for treating progressive MS patients [74]. Myelin production and regeneration need a sufficient number of OPCs to be dispersed appropriately throughout the CNS and differentiation of these progenitor cells into myelin-forming OLs. The precondition for these investigations is the differentiation of stem cells and progenitor cell lines capable of generating oligodendrocytes in culture and modeling the normal brain microenvironment. Due to their permeability, biocompatibility, transparency, inert behavior, similarity to the extracellular matrix (ECM), controllable degradation rate, and adjustable stiffness, hydrogels can be potential matrix to mimic the functional structure of neural tissues and to create a suitable microenvironment for cell growth and proliferation [75].

**Fig. 2:**
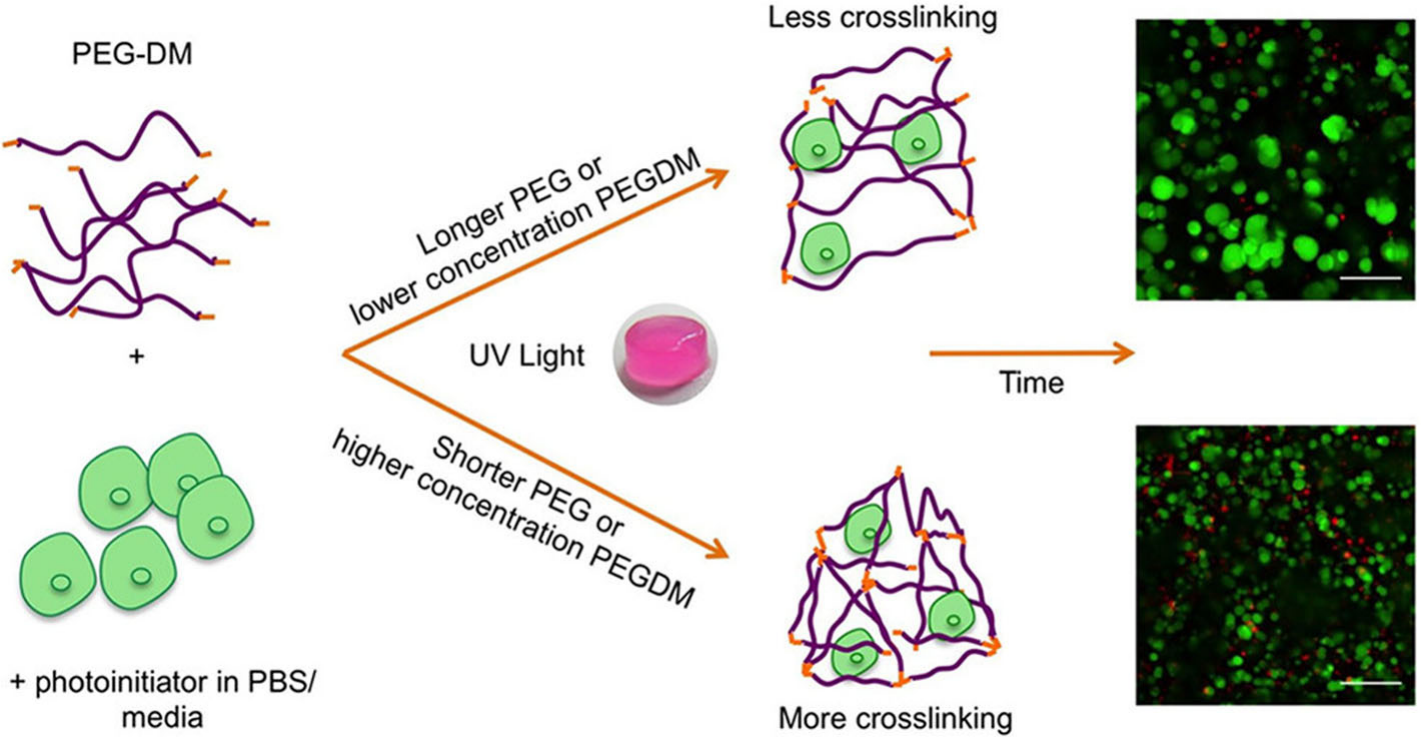
Two encapsulation schemes of oligodendrocyte progenitor cells in PEG-DM hydrogel [78]. Copyright 2017 American Chemical Society

A decade ago, using polyacrylamide hydrogels as two-dimensional culture substrates enabled the establishment of OPCs’ baseline mechanical sensitivity. The findings indicated that the mechanical stiffness of the environment to which these stem cells adhere had a significant effect on their survival, proliferation, migration, and differentiation in vitro. It provides a foundation for investigating the pathological alterations associated with MS and other demyelinating disorders [76]. However, the extracellular environment significantly influences nerve cells’ morphology and electrophysiological property. It is challenging to simulate the characteristics of this type of environment in vitro two-dimensionally. Compared to the monolayer culture of cells, the behavior of cells cultured in a three-dimensional environment is more representative of normal body conditions, necessitating the development of a new generation system to provide a more accurate representation of the intricacy of brain tissue [77]. Russell et al. [78] further investigated the influence of hydrogel characteristics on the survival and proliferation of two different OPC systems enclosed in 3D structures. The results indicated that the two cells’ activity and proliferation depended on the hydrogel’s stiffness and meshed size (see Fig. 2). They stated that this was the first research to establish the impact of hydrogel-mediated proliferation on glia limiting progenitor cells in a three-dimensional environment. These findings show that hydrogels derived from PEG may potentially expand OPCs and control cell destiny in demyelinating illnesses such as MS. Along with their proliferation-promoting properties, elastin-like hydrogels degradable with urokinase plasminogen activator have been shown to stimulate the maturation of oligodendrocyte progenitors, but not enough to differentiate into oligodendrocytes [79]. Meanwhile, Baisiwala et al. established a 3D hydrogel model based on hyaluronic acid that may be utilized to explore the influence of tissue stiffness and inflammation on neural progenitor cell (NPC) development into myelin oligodendrocytes during acute and chronic MS brain damage [80]. A recent piece of research revealed the use of a 3D enzymatically cross-linked gelatin hydrogel system in a microfluidic device to investigate the impact of hypoxia-induced oxidative stress (associated with MS) on the reactivity and myelin sheath of rat glia and human astrocytes [81]. Similarly, there is evidence that 3D HA hydrogel might examine OPC activity, and the low-stiffness microenvironment imitating brain tissue dynamics might promote OPC development and metabolism [82]. Apart from studying the mechanism of gelatin series cells, cultivating oligodendrocytes self-assembling peptide hydrogel system could also yield a conditioned medium rich in neurotrophic factors, which has therapeutic potential in the mouse model of experimental autoimmune encephalomyelitis by preventing demyelinating and glial proliferation [83].

In any case, hydrogel culture is expected to complete the cell adhesion, cytoskeleton, migration, signal transduction, cell differentiation, and morphogenesis of the physical model [84]. Its development is critical to achieve more accurate agents discovered through cell and sensitivity analysis and to investigate the growth and development of cells and tissues in vivo and in vitro mechanisms [85–87]. Organoids and microplatforms based on water coagulation machines aid in the bridge-building between models and clinical practice [88].

### In vivo delivery system

**Fig. 3:**
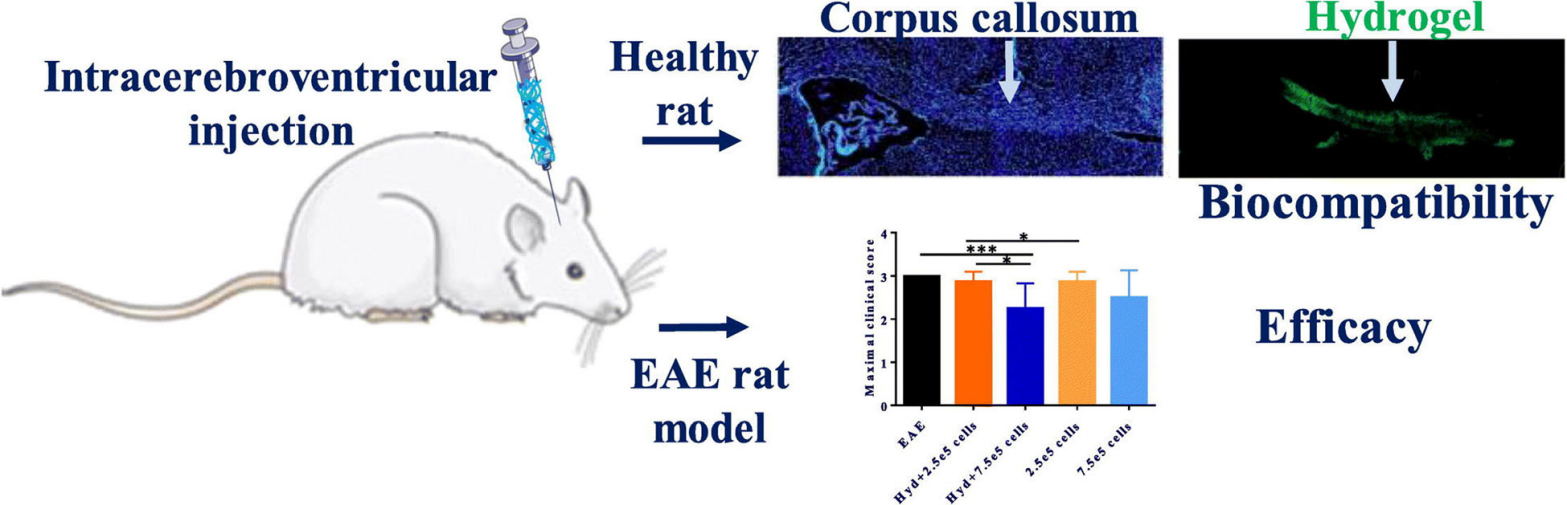
An injectable, biocompatible hydrogel of HA cross-linked with liposome reducing disease severity of EAE [96]. Copyright 2021, with permission from Elsevier

No effective treatments for neurological illnesses such as MS have emerged, but stem cell therapies hold enormous promise for developing novel and curative medicines [45, 89]. Cell transplantation has developed into a nerve injury of cell replacement application, with different cell types transplanted, including human embryonic cells, mesenchymal stem cells derived from human bone marrow and human placenta stem cells, hematopoietic stem cells, human dental pulp stem cells, and undifferentiated adipose stem cells [90]. These cells exhibit anti-inflammatory and immunomodulatory properties, which could significantly slow the progression of experimental autoimmune encephalomyelitis [91]. The majority of research on MS cell treatment does not include NSC since MS cell therapy aims to regulate autoimmune processes rather than to induce myelin repair. While cell treatment has been shown to improve the clinical course of MS significantly, clinical studies have also indicated substantial limits of systemic direct stem cell infusion, including limited cell survival and low central system permeability [92]. Crossing the blood-brain barrier and delivering drugs locally or specifically is a topic that researchers are attempting to tackle. Additionally, encapsulating stem cells in hydrogels or other delivery carriers improves their therapeutic effectiveness significantly [17, 93–95]. Similar mechanical qualities to tissue, excellent biocompatibility, biodegradability, injectability, and porous structure make hydrogels an ideal supportive, protective, and nutritive milieu for cell delivery while avoiding complex invasive surgery [17]. Sustaining stem cell release at particular CNS regions will provide long-term neuroprotective and repair benefits against neurodegenerative disorders, as well as lower delivery dosages. Ferreira et al. [96] recently developed a hyaluronic acid-based hydrogel that was physically cross-linked to liposomes and injected directly into the central nervous system, significantly increasing bone marrow mesenchymal stem cell bioavailability. In EAE models, clinical scores were improved, and neuropathological levels were recovered (see Fig. 3). Apart from neuroprotection and regeneration treatments for MS, dendritic cells (DC), as key actors in immunity, are also potential ways for decreasing the immune response to the myelin sheath [97, 98]. Thomas et al. [99] injected DCs treated with interleukin-10 (IL-10) into cervical lymph nodes using an in situ gel poly (ethylene glycol) based hydrogel. Within 2 days of injection, DCs administration improved the hydrogel’s lifetime and changed the profile of endogenous immune cells recruited at the injection site. Additionally, hydrogels and nanoparticles may be employed as building blocks for more complex nanocomposites, and this technology is especially well suited for intranasal delivery of cells, neuroprotective compounds, and proteins [91, 95].

### Imaging

Clinical impairment in MS and its most often utilized animal model, experimental autoimmune encephalomyelitis (EAE) mice, is linked to gray matter atrophy [100]. Gray matter atrophy, often detected in EAE, has yet to be linked to a specific spinal cord disorder pathology. Clear Lipid-exchanged Acrylamide-hybridized Rigid Imaging-compatible Tissue-hYdrogel or CLARITY is a recently developed optical cleaning technology capable of converting intact tissue into a nanoporous hydrogel hybrid (3-dimensional network crosslinked to hydrophilic polymers) that permits comprehensive imaging of the whole brain with minimum protein loss while keeping natural fluorescence [101, 102]. Using this hydrogel and MRI layer V neurons in EAE mice induced by Thy1.1-YF, Spence et al. [103] demonstrated that cortical volumes correlated negatively to end bulbs and positively to layer V neurons. Later, they used voxel-based morphometry (VBM) to evaluate localized GM atrophy and CLARITY to evaluate specific pathologies in EAE mice. Maps showing the connection between particular diseases and local gray matter atrophy were created [104]. Anatomical insights from these investigations will be useful for future research on gray matter atrophy, which is the strongest predictor of impairment in MS.

Most significantly, the use of MRI and CLARITY to EAE establishes a precedent for better distinguishing neuropathological processes in three-dimensional intact tissues in a range of neurodegenerative illnesses. Another interesting work has shown that by combining the biodegradation of extracellular matrix (ECM) hydrogels with ^19^F MRI of perfluorinated carbon-labeled (PFC) macrophages, novel insights into the participation of neuroinflammatory processes and spatiotemporal dynamics may be obtained. A framework for precise observation of the distribution and density of ^19^F-labeled macrophages in the brain is established by systematic tuning of sequence and imaging parameters. This will further knowledge of peripheral macrophages’ participation in bio-scaffold breakdown and regeneration of brain tissue [105]. These investigations of macrophages as pathogenic cell effectors and prospective therapeutic targets in MS [106], in conjunction with labeled imaging and hydrogels, present a potential in vivo tool to aid in the monitoring and prevention of MS. Simultaneously, hydrogels may be turned into fluorescent probes for cell imaging, allowing for fast diagnosis of neurodegenerative biomarkers [107].

## Conclusions and outlook

MS requires additional therapies since it is a progressive impairment. Analyses of the complete spectrum of hydrogel-related MS diagnostic and treatment techniques generate several research interests. For example, the intestinal tract microbiome is a rapidly growing area of research into the pathogenesis and potential treatments of MS, as peripheral immune activation plays a role in the disease’s pathogenesis and a symbiotic intestinal tract microbiome may be necessary for initiating the immune response [108]. Additionally, this review suggests that the delivery of combining hydrogels with intestinal bacteria that are resistant to stomach acid and decompose on demand may be a future research area [109]. Another area of interest for future hydrogel applications is wearable electronic sensors, which have the potential to achieve volume production while delivering many beneficial features such as monitoring balance, tiredness, and movement throughout the course of MS. More intriguingly, 3D hydrogel systems facilitate the cultivation and elucidation of cell-matrix interactions by reproducing the unique properties of native central nervous system tissues in vitro, including binding delivery through nanoparticles. These may be the areas of attention for the development of hydrogel materials, as well as for MS diagnosis and prognosis monitoring.

## Data Availability

All data generated or analyzed during this study are included in this published article and its supplementary information files.

## References

[1] Cerexhe L, Easton C, Macdonald E, Renfrew L, Sculthorpe N (2022). Blood lactate concentrations during rest and exercise in people with multiple sclerosis: a systematic review and meta-analysis. Mult Scler Relat Disord.

[2] Dobson R, Giovannoni G (2019). Multiple sclerosis – a review. Eur J Neurol.

[3] Carotenuto A, Valsasina P, de la Cruz MH, Cacciaguerra L, Preziosa P, Marchesi O, et al. Divergent time-varying connectivity of thalamic sub-regions characterizes clinical phenotypes and cognitive status in multiple sclerosis. Mol Psychiatry. 2022. 10.1038/s41380-021-01401-w 34992237

[4] Kolbe SC, Garcia LM, Yu N, Boonstra FM, Dough M, Sinclair B (2022). Lesion volume in relapsing multiple sclerosis is associated with perivascular space enlargement at the level of the basal ganglia. Am J Neuroradiol.

[5] Virgilio E, Vecchio D, Crespi I, Puricelli C, Barbero P, Galli G, et al. Cerebrospinal fluid biomarkers and cognitive functions at multiple sclerosis diagnosis. J Neurol. 2022. 10.1007/s00415-021-10945-4 35088141

[6] Solomon AJ, Klein EP, Bourdette D (2012). “Undiagnosing” multiple sclerosis the challenge of misdiagnosis in MS. Neurology..

[7] The Lancet Magazine. End of the road for daclizumab in multiple sclerosis. Lancet. 2018;391(10125):1000. 10.1016/S0140-6736(18)30565-8 29565004

[8] Metz LM, Liu W-Q (2018). Effective treatment of progressive MS remains elusive. Lancet..

[9] Atkins HL, Bowman M, Allan D, Anstee G, Arnold DL, Bar-Or A (2016). Immunoablation and autologous haemopoietic stem-cell transplantation for aggressive multiple sclerosis: a multicentre single-group phase 2 trial. Lancet..

[10] Annabi N, Tamayol A, Uquillas JA, Akbari M, Bertassoni LE, Cha C (2014). 25th anniversary article: rational design and applications of hydrogels in regenerative medicine. Adv Mater.

[11] Barrett-Catton E, Ross ML, Asuri P (2021). Multifunctional hydrogel nanocomposites for biomedical applications. Polymers..

[12] Chyzy A, Plonska-Brzezinska ME (2020). Hydrogel properties and their impact on regenerative medicine and tissue engineering. Molecules..

[13] Li J, Wu C, Chu PK, Gelinsky M (2020). 3D printing of hydrogels: rational design strategies and emerging biomedical applications. Mater Sci Eng R-Reports.

[14] Zhang K, Gao G, Li Y, Song Y, Wen Y, Zhang X (2021). Development and application of DNA hydrogel in biosensing. Prog Chem.

[15] Jiang Y, Krishnan N, Heo J, Fang RH, Zhang L (2020). Nanoparticle–hydrogel superstructures for biomedical applications. J Control Release.

[16] Lin PH, Dong Q, Chew SY (2021). Injectable hydrogels in stroke and spinal cord injury treatment: a review on hydrogel materials, cell–matrix interactions and glial involvement. Mater Adv.

[17] Jarrin S, Cabré S, Dowd E (2021). The potential of biomaterials for central nervous system cellular repair. Neurochem Int.

[18] Caló E, Khutoryanskiy VV (2015). Biomedical applications of hydrogels: a review of patents and commercial products. Eur Polym J.

[19] Hamidi M, Azadi A, Rafiei P (2008). Hydrogel nanoparticles in drug delivery. Adv Drug Deliv Rev.

[20] Le X, Lu W, Zhang J, Chen T (2019). Recent Progress in biomimetic anisotropic hydrogel actuators. Adv Sci.

[21] Sheng H, Xue B, Qin M, Wang W, Cao Y (2020). Preparation and applications of stretchable and tough hydrogels. Chem J Chinese Univ-Chinese.

[22] Song P, Ye D, Song S, Wang L, Zuo X (2016). Preparation and biological applications of DNA hydrogel. Prog Chem.

[23] Su X, Ge C, Chen L, Xu Y (2020). Hydrogel-based sensing detection of Bacteria. Prog Chem.

[24] Xiao Y, Gu Y, Qin L, Chen L, Chen X, Cui W (2021). Injectable thermosensitive hydrogel-based drug delivery system for local cancer therapy. Colloids Surf B-Biointerfaces.

[25] Xu J, Tsai Y-L, Hsu S-h (2020). Design strategies of conductive hydrogel for biomedical applications. Molecules..

[26] Yue S, He H, Li B, Hou T (2020). Hydrogel as a Biomaterial for Bone Tissue Engineering: A Review. Nanomaterials (Basel).

[27] Owens B (2016). Multiple sclerosis. Nature..

[28] Briggs FBS, Hill E (2019). Estimating the prevalence of multiple sclerosis using 56.6 million electronic health records from the United States. Mult Scler J.

[29] Tintore M, Vidal-Jordana A, Sastre-Garriga J (2019). Treatment of multiple sclerosis — success from bench to bedside. Nat Rev Neurol.

[30] Ryan L, Mills KHG. Sex differences regulate immune responses in experimental autoimmune encephalomyelitis and multiple sclerosis.Eur J Immunol. 2022;52(1):24–33. 10.1002/eji.202149589 34727577

[31] Gourraud P-A, Harbo HF, Hauser SL, Baranzini SE (2012). The genetics of multiple sclerosis: an up-to-date review. Immunol Rev.

[32] Olsson T, Barcellos LF, Alfredsson L (2017). Interactions between genetic, lifestyle and environmental risk factors for multiple sclerosis. Nat Rev Neurol.

[33] Kamm CP, Uitdehaag BM, Polman CH (2014). Multiple sclerosis: current knowledge and future outlook. Eur Neurol.

[34] Thompson AJ, Baranzini SE, Geurts J, Hemmer B, Ciccarelli O (2018). Multiple sclerosis. Lancet.

[35] Tabansky I, Messina MD, Bangeranye C, Goldstein J, Blitz-Shabbir KM, Machado S (2015). Advancing drug delivery systems for the treatment of multiple sclerosis. Immunol Res.

[36] Trapp BD, Peterson J, Ransohoff RM, Rudick R, Mörk S, Bö L (1998). Axonal transection in the lesions of multiple sclerosis. N Engl J Med.

[37] Segal BM, Stüve O (2016). Primary progressive multiple sclerosis—why we are failing. Lancet.

[38] Cavanagh JJ, Levy M. Differential diagnosis of multiple sclerosis. Presse medicale (Paris, France : 1983). 2021:104092-. 10.1016/j.lpm.2021.104092 34715293

[39] Brownlee WJ, Hardy TA, Fazekas F, Miller DH (2017). Diagnosis of multiple sclerosis: progress and challenges. Lancet.

[40] Thompson AJ, Banwell BL, Barkhof F, Carroll WM, Coetzee T, Comi G (2018). Diagnosis of multiple sclerosis: 2017 revisions of the McDonald criteria. Lancet Neurol.

[41] Rovira À, Wattjes MP, Tintoré M, Tur C, Yousry TA, Sormani MP (2015). MAGNIMS consensus guidelines on the use of MRI in multiple sclerosis—clinical implementation in the diagnostic process. Nat Rev Neurol.

[42] Dolati S, Babaloo Z, Jadidi-Niaragh F, Ayromlou H, Sadreddini S, Yousefi M (2017). Multiple sclerosis: therapeutic applications of advancing drug delivery systems. Biomed Pharmacother.

[43] Mukherjee N, Adak A, Ghosh S (2020). Recent trends in the development of peptide and protein-based hydrogel therapeutics for the healing of CNS injury. Soft Matter.

[44] Singh AV, Chandrasekar V, Janapareddy P, Mathews DE, Laux P, Luch A (2021). Emerging application of nanorobotics and artificial intelligence to cross the BBB: advances in design, controlled maneuvering, and targeting of the barriers. ACS Chem Neurosci.

[45] Oliveira AG, Gonçalves M, Ferreira H, Neves NM (2020). Growing evidence supporting the use of mesenchymal stem cell therapies in multiple sclerosis: a systematic review. Mult Scler Relat Disord.

[46] Korshoj LE, Kielian T (2021). Neuroimmune metabolism: uncovering the role of metabolic reprogramming in central nervous system disease. J Neurochem.

[47] Shimizu K, Agata K, Takasugi S, Goto S, Narita Y, Asai T (2021). New strategy for MS treatment with autoantigen-modified liposomes and their therapeutic effect. J Control Release.

[48] Kwiatkowski AJ, Stewart JM, Cho JJ, Avram D, Keselowsky BG (2020). Nano and Microparticle emerging strategies for treatment of autoimmune diseases: multiple sclerosis and type 1 diabetes. Adv Healthcare Mater.

[49] Boesen F, Nørgaard M, Trénel P, Rasmussen PV, Petersen T, Løvendahl B (2017). Longer term effectiveness of inpatient multidisciplinary rehabilitation on health-related quality of life in MS patients: a pragmatic randomized controlled trial – the Danish MS hospitals rehabilitation study. Mult Scler J.

[50] Fu L, Yu A, Lai G (2021). Conductive hydrogel-based electrochemical sensor: a soft platform for capturing Analyte. Chemosensors..

[51] Fu L, Liu Z, Ge J, Guo M, Zhang H, Chen F (2019). (001) plan manipulation of α-Fe2O3 nanostructures for enhanced electrochemical Cr(VI) sensing. J Electroanal Chem.

[52] Abune L, Davis B, Wang Y (2021). Aptamer-functionalized hydrogels: an emerging class of biomaterials for protein delivery, cell capture, regenerative medicine, and molecular biosensing. Wiley Interdiscip Rev Nanomed Nanobiotechnol.

[53] Avolio C, Ruggieri M, Giuliani F, Liuzzi GM, Leante R, Riccio P (2003). Serum MMP-2 and MMP-9 are elevated in different multiple sclerosis subtypes. J Neuroimmunol.

[54] Benešová Y, Vašků A, Novotná H, Litzman J, Štourač P, Beránek M (2009). Matrix metalloproteinase-9 and matrix metalloproteinase-2 as biomarkers of various courses in multiple sclerosis. Mult Scler J.

[55] Dhanjai SA, Kalambate PK, Mugo SM, Kamau P, Chen J (2019). Polymer hydrogel interfaces in electrochemical sensing strategies: a review. Trac-trends Anal Chem.

[56] Wang R, Li Y (2013). Hydrogel based QCM aptasensor for detection of avian influenza virus. Biosens Bioelectron.

[57] Stair JL, Watkinson M, Krause S (2009). Sensor materials for the detection of proteases. Biosens Bioelectron.

[58] Biela A, Watkinson M, Meier UC, Baker D, Giovannoni G, Becer CR (2015). Disposable MMP-9 sensor based on the degradation of peptide cross-linked hydrogel films using electrochemical impedance spectroscopy. Biosens Bioelectron.

[59] Ahmad N, Colak B, Gibbs MJ, Zhang D-W, Gautrot JE, Watkinson M (2019). Peptide cross-linked poly(2-oxazoline) as a sensor material for the detection of proteases with a quartz crystal microbalance. Biomacromolecules..

[60] Zhu Y, Haghniaz R, Hartel MC, Mou L, Tian X, Garrido PR, et al. Recent advances in bioinspired hydrogels: materials, Devices, and Biosignal Computing. ACS Biomater Sci Eng. 2021. 10.1021/acsbiomaterials.1c00741 PMC10823919 34784170

[61] Rahmani P, Shojaei A (2021). A review on the features, performance and potential applications of hydrogel-based wearable strain/pressure sensors. Adv Colloid Interf Sci.

[62] Agrawal M, Prathyusha E, Ahmed H, Dubey SK, Kesharwani P, Singhvi G (2021). Biomaterials in treatment of Alzheimer’s disease. Neurochem Int.

[63] Wang Z, Liu Y, Wang Z, Huang X, Huang W (2021). Hydrogel-based composites: Unlimited platforms for biosensors and diagnostics. View.

[64] Herrmann A, Haag R, Schedler U (2021). Hydrogels and Their Role in Biosensing Applications. Adv Healthc Mater.

[65] Li S, Zhou H, Li Y, Jin X, Liu H, Lai J (2022). Mussel-inspired self-adhesive hydrogels by conducting free radical polymerization in both aqueous phase and micelle phase and their applications in flexible sensors. J Colloid Interface Sci.

[66] Li S-N, Yu Z-R, Guo B-F, Guo K-Y, Li Y, Gong L-X (2021). Environmentally stable, mechanically flexible, self-adhesive, and electrically conductive Ti3C2TX MXene hydrogels for wide-temperature strain sensing. Nano Energy.

[67] Liang Y, Shen Y, Sun X, Liang H (2021). Preparation of stretchable and self-healable dual ionically cross-linked hydrogel based on chitosan/polyacrylic acid with anti-freezing property for multi-model flexible sensing and detection. Int J Biol Macromol.

[68] Lu C, Qiu J, Zhao W, Sakai E, Zhang G. A tough hydrogel with fast self-healing and adhesive performance for wearable sensors. Colloids Surf a-Physicochem EngAspects. 2022;632.

[69] Meyer BM, Tulipani LJ, Gurchiek RD, Allen DA, Adamowicz L, Larie D (2021). Wearables and deep learning classify fall risk from gait in multiple sclerosis. Ieee J Biomed Health Informatics.

[70] Mueller R, Hamacher D, Hansen S, Oschmann P, Keune PM (2021). Wearable inertial sensors are highly sensitive in the detection of gait disturbances and fatigue at early stages of multiple sclerosis. BMC Neurol.

[71] Monschein T, Leutmezer F, Altmann P (2021). The use of wearable devices in multiple sclerosis. Klinische Neurophysiologie.

[72] Kim J-N, Lee J, Lee H, Oh I-K (2021). Stretchable and self-healable catechol-chitosan-diatom hydrogel for triboelectric generator and self-powered tremor sensor targeting at Parkinson disease. Nano Energy.

[73] Hasanzadeh M, Shadjou N, de la Guardia M (2014). Electrochemical biosensing using hydrogel nanoparticles. TrAC Trends Anal Chem.

[74] Hauser SL, Chan JR, Oksenberg JR (2013). Multiple sclerosis: Prospects and promise. Ann Neurol.

[75] Zhou P, Xu P, Guan J, Zhang C, Chang J, Yang F (2020). Promoting 3D neuronal differentiation in hydrogel for spinal cord regeneration. Colloids Surf B: Biointerfaces.

[76] Jagielska A, Norman AL, Whyte G, Vliet KJV, Guck J, Franklin RJM (2012). Mechanical environment modulates biological properties of oligodendrocyte progenitor cells. Stem Cells Dev.

[77] Liu H, Wang Y, Cui K, Guo Y, Zhang X, Qin J (2019). Advances in hydrogels in organoids and organs-on-a-Chip. Adv Mater.

[78] Russell LN, Lampe KJ (2017). Oligodendrocyte precursor cell viability, proliferation, and morphology is dependent on mesh size and storage Modulus in 3D poly(ethylene glycol)-based hydrogels. ACS Biomaterials Science & Engineering.

[79] Meco E, Zheng WS, Sharma AH, Lampe KJ (2020). Guiding oligodendrocyte precursor cell maturation with Urokinase plasminogen activator-degradable elastin-like protein hydrogels. Biomacromolecules..

[80] Baisiwala S, Moreno MA, Wang C, Rogan HAW, Tsai H-C, Yang F (2017). A 3-dimensional hydrogel model of multiple sclerosis brain lesions reveals insights into re-myelination. J Biomater Tissue Eng.

[81] Zambutot SG, Serranot JF, Vilbert AC, Lu Y, Harley BAC, Pedron S (2020). Response of neuroglia to hypoxia-induced oxidative stress using enzymatically crosslinked hydrogels. MRS Commun.

[82] Unal DB, Caliari SR, Lampe KJ (2020). 3D hyaluronic acid hydrogels for modeling oligodendrocyte progenitor cell behavior as a function of matrix stiffness. Biomacromolecules..

[83] Jahanbazi Jahan-Abad A, Karima S, Sahab Negah S, Noorbakhsh F, Borhani-Haghighi M, Gorji A (2019). Therapeutic potential of conditioned medium derived from oligodendrocytes cultured in a self-assembling peptide nanoscaffold in experimental autoimmune encephalomyelitis. Brain Res.

[84] Schindler M, Nur-E-Kamal A, Ahmed I, Kamal J, Liu H-Y, Amor N (2006). Living in three dimensions. Cell Biochem Biophys.

[85] Argentiere S, Siciliano PA, Blasi L (2021). How microgels can improve the impact of organ-on-Chip and Microfluidic devices for 3D culture: compartmentalization, Single Cell Encapsulation and Control on Cell Fate. Polymers.

[86] Morales X, Cortes-Dominguez I, Ortiz-de-Solorzano C (2021). Modeling the Mechanobiology of Cancer cell migration using 3D biomimetic hydrogels. Gels..

[87] Namgung B, Ravi K, Vikraman PP, Sengupta S, Jang HL (2021). Engineered cell-laden alginate microparticles for 3D culture. Biochem Soc Trans.

[88] Yi Y, Park J, Lim J, Lee CJ, Lee S-H (2015). Central nervous system and its disease models on a Chip. Trends Biotechnol.

[89] Martino G, Franklin RJM, Van Evercooren AB, Kerr DA (2010). The stem cells in multiple sclerosis consensus G. stem cell transplantation in multiple sclerosis: current status and future prospects. Nat Rev Neurol.

[90] Scolding NJ, Pasquini M, Reingold SC, Cohen JA, Sclerosis: ICoC-BTfM (2017). Cell-based therapeutic strategies for multiple sclerosis. Brain..

[91] Matías-Guiu J, Matías-Guiu JA, Montero-Escribano P, Barcia JA, Canales-Aguirre AA, Mateos-Diaz JC (2020). Particles Containing Cells as a Strategy to Promote Remyelination in Patients With Multiple Sclerosis. Front Neurol.

[92] Uccelli A, Laroni A, Freedman MS (2011). Mesenchymal stem cells for the treatment of multiple sclerosis and other neurological diseases. Lancet Neurol.

[93] Albani D, Gloria A, Giordano C, Rodilossi S, Russo T, D’Amora U (2013). Hydrogel-based nanocomposites and mesenchymal stem cells: a promising synergistic strategy for neurodegenerative disorders therapy. Sci World J.

[94] Giordano C, Albani D, Gloria A, Tunesi M, Batelli S, Russo T (2009). Multidisciplinary perspectives for Alzheimer’s and Parkinson’s diseases: hydrogels for protein delivery and cell-based drug delivery as therapeutic strategies. Int J Artif Organs.

[95] Giordano C, Albani D, Gloria A, Tunesi M, Rodilossi S, Russo T (2011). Nanocomposites for neurodegenerative diseases: hydrogel-nanoparticle combinations for a challenging drug delivery. Int J Artif Organs.

[96] Ferreira H, Amorim D, Lima AC, Pirraco RP, Costa-Pinto AR, Almeida R (2021). A biocompatible and injectable hydrogel to boost the efficacy of stem cells in neurodegenerative diseases treatment. Life Sci.

[97] Greter M, Heppner FL, Lemos MP, Odermatt BM, Goebels N, Laufer T (2005). Dendritic cells permit immune invasion of the CNS in an animal model of multiple sclerosis. Nat Med.

[98] Karni A, Abraham M, Monsonego A, Cai G, Freeman GJ, Hafler D (2006). Innate immunity in multiple sclerosis: myeloid dendritic cells in secondary progressive multiple sclerosis are activated and drive a Proinflammatory immune response. J Immunol.

[99] Thomas AM, Beskid NM, Blanchfield JL, Rosado AM, García AJ, Evavold BD (2021). Localized hydrogel delivery of dendritic cells for attenuation of multiple sclerosis in a murine model. J Biomed Mater Res A.

[100] Chard D, Miller D (2009). Grey matter pathology in clinically early multiple sclerosis: evidence from magnetic resonance imaging. J Neurol Sci.

[101] Chung K, Wallace J, Kim S-Y, Kalyanasundaram S, Andalman AS, Davidson TJ (2013). Structural and molecular interrogation of intact biological systems. Nature..

[102] Tomer R, Ye L, Hsueh B, Deisseroth K (2014). Advanced CLARITY for rapid and high-resolution imaging of intact tissues. Nat Protoc.

[103] Spence RD, Kurth F, Itoh N, Mongerson CRL, Wailes SH, Peng MS (2014). Bringing clarity to gray matter atrophy. NeuroImage..

[104] Meyer CE, Gao JL, Cheng JY-J, Oberoi MR, Johnsonbaugh H, Lepore S (2019). Axonal damage in spinal cord is associated with gray matter atrophy in sensorimotor cortex in experimental autoimmune encephalomyelitis. Mult Scler J.

[105] Ghuman H, Hitchens TK, Modo M (2019). A systematic optimization of 19F MR image acquisition to detect macrophage invasion into an ECM hydrogel implanted in the stroke-damaged brain. NeuroImage..

[106] Mammana S, Fagone P, Cavalli E, Basile MS, Petralia MC, Nicoletti F (2018). The role of macrophages in Neuroinflammatory and neurodegenerative pathways of Alzheimer’s disease, amyotrophic lateral sclerosis, and multiple sclerosis: Pathogenetic cellular effectors and potential therapeutic targets. Int J Mol Sci.

[107] Hamd-Ghadareh S, Salimi A, Parsa S, Mowla SJ (2022). Development of three-dimensional semi-solid hydrogel matrices for ratiometric fluorescence sensing of amyloid β peptide and imaging in SH-SY5 cells: improvement of point of care diagnosis of Alzheimer’s disease biomarker. Biosens Bioelectron.

[108] Zhu W, Dykstra K, Zhang L, Xia Z (2021). Gut microbiome as potential therapeutics in multiple sclerosis. Curr Treat Options Neurol.

[109] Kim J, Hlaing SP, Lee J, Saparbayeva A, Kim S, Hwang DS (2021). Exfoliated bentonite/alginate nanocomposite hydrogel enhances intestinal delivery of probiotics by resistance to gastric pH and on-demand disintegration. Carbohydr Polym.

